# A Pilot Study of *Klebsiella pneumoniae* in Community-Acquired Pneumonia: Comparative Insights from Culture and Targeted Next-Generation Sequencing

**DOI:** 10.3390/diagnostics16010154

**Published:** 2026-01-04

**Authors:** Vyacheslav Beloussov, Vitaliy Strochkov, Nurlan Sandybayev, Alyona Lavrinenko, Maxim Solomadin

**Affiliations:** 1 Molecular Genetic Laboratory TreeGene, Almaty 050008, Kazakhstan; 2 Kazakhstan-Japan Innovation Centre, Kazakh National Agrarian Research University, Almaty 050010, Kazakhstan; nurlan.s@kaznaru.edu.kz; 3 Scientific Research Laboratory, Karaganda Medical University, Karaganda 100000, Kazakhstan; lavrinenko@qmu.kz (A.L.); maks50@gmail.com (M.S.)

**Keywords:** community-acquired pneumonia, *Klebsiella pneumoniae*, MALDI-TOF MS, DDM, AMR, tNGS, ARGs, resistome

## Abstract

**Background/Objectives**: *Klebsiella pneumoniae* is a major Gram-negative pathogen associated with community-acquired pneumonia (CAP) and a critical contributor to antimicrobial resistance (AMR). Culture-based diagnostics remain the clinical standard but may underestimate microbial diversity and resistance gene profiles. This pilot study compared pathogen detection and antimicrobial resistance gene (ARG) repertoires in matched *K. pneumoniae* pure cultures and primary sputum samples using targeted next-generation sequencing (tNGS). **Methods**: We analyzed 153 sputum samples from patients with CAP. Among 48 culture-positive cases, 22 (14% overall; 54% culture-positive) yielded *K. pneumoniae*. MALDI-TOF MS, phenotypic drug susceptibility testing, and tNGS were conducted on both culture isolates and matched sputum specimens. Microbial composition, ARG diversity, and method concordance were evaluated, with focused analysis of discordant and fatal cases. **Results**: *K. pneumoniae* was detected in 14.4% of all CAP cases and accounted for 54.2% of culture-positive samples. Identification rates differed across methods: 35% by MALDI-TOF MS, 45% by culture tNGS, and 29% by sputum tNGS. Sputum tNGS revealed substantially higher microbial diversity than cultures (3.04 vs. 1.42 species per sample) and detected more than sixfold unique ARGs (38 vs. 7), including clinically relevant determinants that were absent from culture isolates. Concordance was high between MALDI-TOF MS and culture tNGS (κ = 0.712), but low between sputum and culture tNGS (κ = 0.279). Among twelve *K. pneumoniae* isolates included in AMR analysis, all showed resistance to β-lactams, and two-thirds exhibited MDR/XDR phenotypes. Genotypic screening identified seven ARGs, but major ESBL and carbapenemase genes were not detected, suggesting the presence of alternative resistance mechanisms. Overall, sputum tNGS provided additional etiological and resistome information not captured by cultivation and complemented classical diagnostics in CAP involving *K. pneumoniae*. **Conclusions**: Culture-based diagnostics and tNGS provide complementary insights into the detection and resistance profiling of *K. pneumoniae* in CAP, with sputum tNGS revealing broader microbial and resistome information than pure cultures, while classical methods remain essential for species confirmation and phenotypic AST. An integrated diagnostic approach combining both methodologies may improve pathogen detection, guide antimicrobial therapy, and enhance AMR surveillance in *K. pneumoniae*-associated CAP.

## 1. Introduction

Community-acquired pneumonia (CAP) remains a major global health problem, leading to significant morbidity, mortality, and healthcare burden [1]. It typically presents with symptoms such as fever, cough with sputum, dyspnea, and chest pain, and is most common in older adults, smokers, and patients with chronic comorbidities. The etiological spectrum is wide, including bacterial pathogens (e.g., *Streptococcus pneumoniae*, *Haemophilus influenzae*, *Klebsiella pneumoniae*), viruses (e.g., influenza and RSV), and atypical agents (e.g., *Mycoplasma pneumoniae* and *Chlamydophila pneumoniae*). This diversity underscores the need for advanced diagnostic and therapeutic strategies [2].

*K. pneumoniae*, a Gram-negative opportunistic pathogen from the *Enterobacteriaceae* family, is one of the infections that can cause CAP [3,4,5]. This organism has a strong association with the resident human flora and colonizes nearly all parts of the human body, predominantly in the respiratory, gastrointestinal, and urinary tracts [6]. Moreover, it is one of the leading causative agents of nosocomial infections, belonging to the group of so-called “ESKAPE” pathogens, microorganisms associated with increased antibiotic resistance [7,8], and posing a serious problem for healthcare [9,10,11], increasing mortality in severely ill patients [12], and harming financial costs during hospitalization [13].

While traditional culture methods remain the standard approach for identifying bacterial respiratory infections [14], they face limitations, including cultivation challenges and prolonged turnaround times [15]. Alternative diagnostic techniques, including mass spectrometry, real-time PCR, and immunoassays, have also been utilized for laboratory diagnosis, but their capabilities are restricted to detecting a limited range of microorganisms [16]. To address these limitations, next-generation sequencing (NGS) has emerged as a promising alternative that can provide a comprehensive profile of the microbial communities in clinical samples [17,18,19,20]. This technique allows for the simultaneous detection and identification of a wide range of pathogens, including rare or unculturable organisms, and can provide insights into antimicrobial resistance genes (ARGs) [16,21].

Targeted NGS (tNGS) approaches have shown promise in improving the accuracy of AMR detection in patients with bacterial pneumonia [22,23,24]. These methods also offer rapid and sensitive detection of ARGs, which is crucial for timely, effective treatment. The integration of genomic data with clinical diagnostics can enhance the prediction and identification of AMR, providing a more comprehensive understanding of resistance patterns [25]. tNGS panels can detect a wide range of pathogens and ARGs, facilitating large-scale surveillance and providing insights into the epidemiology of resistance genes across different regions [26]. tNGS offers several advantages for detecting ARGs in *K. pneumoniae*, including rapid identification of a wide range of ARGs, improved sensitivity and specificity compared to traditional methods, faster turnaround time, and enhanced regional and global surveillance of resistance patterns [27].

While tNGS approaches offer significant advantages in AMR detection, challenges remain, including the need for standardized protocols and integration into routine clinical practice. Additionally, the cost and complexity of tNGS may limit its widespread adoption, necessitating further research and development to optimize these technologies for broader clinical use [28].

In this pilot study, we performed an integrated analysis of primary sputum samples and corresponding bacterial isolates from patients with CAP to characterize the contribution of *K. pneumoniae* and its antimicrobial resistance patterns. Using conventional culture, MALDI-TOF MS, disk diffusion method (DDM), and tNGS, we compared diagnostic outputs across methods to assess their concordance and limitations. This approach aimed to clarify the added value of molecular techniques alongside classical workflows and to improve the understanding of pathogen detection and resistance profiling in CAP.

## 2. Materials and Methods

### 2.1. Study Design

A pilot, prospective, cohort study, which was conducted collaboratively at two institutions: Karaganda Medical University and Kazakh National Agrarian Research University. The inclusion criteria were adult age (≥18 years), clinical signs of CAP, absence of antibiotic use within the preceding two weeks, and successful culture growth of a presumptive CAP pathogen. The exclusion criteria were sputum samples without bacterial growth, those yielding only normal oropharyngeal flora by culture and MALDI-TOF MS, and samples with insufficient volume.

Inclusion was limited to culture-positive cases in which *K. pneumoniae* was detected by all three diagnostic methods, ensuring strict method-to-method comparability. Although this approach provided methodological consistency, it inherently biased the cohort toward cultivable organisms, potentially underrepresenting fastidious or non-culturable pathogens detectable only by molecular techniques.

### 2.2. Sample Collection and Preparation

Sputum samples were collected from adult patients with clinical signs of CAP at three hospitals in Karaganda, Kazakhstan: The Clinic at the Medical University, The Hematology Center, and The Cardiology Center. Samples were obtained within 48 h of hospital admission to exclude cases of hospital-acquired pneumonia (HAP). Collection and transport of materials were performed in accordance with the sanitary standards of the Republic of Kazakhstan. All patients completed structured questionnaires, and written informed consent was obtained prior to inclusion in this study.

### 2.3. Microbiological Methods

Sputum was collected in sterile containers following thorough patient instruction. Sample collection was performed in the morning. Patients were advised to perform oral hygiene, then take a deep breath and cough vigorously to obtain sputum from the lower respiratory tract, while avoiding contamination with saliva [29]. Subsequently, the samples were transported to the research laboratory within 2 h of collection. Sample cultivation was performed on blood agar supplemented with 5% defibrinated sheep blood (ThermoFisher, Carlsbad, CA, USA) and Sabouraud medium [30]. The cultures were incubated at 37 °C for 24–48 h under aerobic conditions. Additionally, plates were incubated in an atmosphere of 5% CO_2_ for the cultivation of fastidious microorganisms (*Haemophilus* spp., *Streptococcus* spp.) [31].

For microorganism identification, fresh colonies were used. The direct spotting method was applied: a small amount of bacterial mass was picked up with a loop tip and applied as a thin film onto a steel target plate. The sample was then covered with 1 μL of the matrix solution (α-cyano-4-hydroxycinnamic acid) and air-dried at room temperature [32]. Bacterial isolates were identified to the species level using MALDI-TOF MS (Microflex LT, Bruker Daltonics, Hamburg, Germany) with MALDI Biotyper Compass 4.1.80; scores ≥ 2.2 indicated reliable identification [33,34]. Isolates were stored at −70 °C in trypticase soy broth containing 30% glycerol until testing. Antimicrobial susceptibility testing (AST) was performed by disk diffusion on Mueller-Hinton agar according to EUCAST v 13.1; colistin was interpreted per CLSI M100 ED33. *E. coli* ATCC^®^ 25922, *E. coli* ATCC^®^ 35218, and *P. aeruginosa* ATCC^®^ 27853 served as quality control strains. AST results were entered into AMRmap [35]. Resistance classification followed ECDC/CDC definitions [36]: AMR—non-susceptibility to ≥1 agent in any class; MDR—non-susceptibility to ≥1 agent in ≥3 classes; XDR—non-susceptibility to all but ≤2 classes; PDR—non-susceptibility to all agents in all classes.

### 2.4. DNA Extraction and Quality Assessment

DNA was isolated from 200 µL of pure bacterial culture and from each patient’s sputum using a PureLink Microbiome DNA Purification kit (Thermo Fisher Scientific, Carlsbad, CA, USA) according to the manufacturer’s instructions. The isolated DNA was stored in a freezer at −20 °C and used as intended.

The quality of isolated DNA was checked spectrophotometrically using a NanoDrop 2000, and DNA concentration was measured on a Qubit 3.0 Fluorimeter using the Qubit 1× dsDNA HS Assay kit (Thermo Fisher Scientific, Eugen, OR, USA), according to the manufacturer’s instructions. DNA purity was assessed by absorbance ratios; A260/280 values of 1.8–2.0 were considered indicative of pure DNA, and A260/230 values of 2.0–2.2 were acceptable for downstream molecular analyses.

### 2.5. Preparing Libraries and Tngs

Genomic DNA (10 ng) was processed using the Ion AmpliSeq™ Pan-Bacterial Research Panel and AmpliSeq Kit v2.0 (Thermo Fisher Scientific, Carlsbad, CA, USA) according to the manufacturer’s protocol. The panel, developed with Lawrence Livermore National Laboratory (USA), enables 16S rRNA-based genus/species identification and detection of ARGs from 31 antibiotic classes via two primer pools (269 amplicons for 21 bacterial species; 716 amplicons for 364 ARGs). Libraries were adapter-ligated, purified, quantified (Ion Library TaqMan^®^ Quantitation Kit, ThermoFisher, Vilnius, Lithuania), pooled, and sequenced on the Ion S5 with Ion Chef (ThermoFisher, Waltham, MA, USA) using Ion 540™ chips and sequencing kits (ThermoFisher, Carlsbad, CA, USA). Data processing on the Ion Torrent Server v. 5.18 included quality filtering, size selection, and removal of polyclonal reads/adapter dimers, with final outputs in FASTQ format.

### 2.6. Bioinformatics Analysis

tNGS data were processed using Ion Reporter v.5.18 software (Thermo Fisher Scientific, USA) for taxonomic profiling based on 16S rRNA sequences (genus and species level) and for ARG detection using the integrated ResFinder database. Each sample was analyzed using unique barcodes on either the One Codex web platform “https://app.onecodex.com (accessed on 20 June 2025)” for ARG profiling with the AMR Research Panel, reporting ARG coverage, identity to reference sequences, and read depth, or the PanBacterialAnalysis plugin on the Ion Torrent Server for Pan-Bacterial Research Panel data, reporting read counts per ARG.

The Normalized Read Count for a species or gene was calculated as the ratio of the sum of read counts across all amplicons mapping to that target to the total reads in the sample, with a default detection threshold of >0.01. For AMR genes, the detection threshold was 0.1% of total reads, in accordance with the manufacturer’s recommendations. The Comprehensive Antibiotic Resistance Database (CARD; https://card.mcmaster.ca/ accessed on 20 June 2025) was additionally used to annotate detected ARGs, refine mechanism-level interpretation, and classify resistance determinants according to the Antibiotic Resistance Ontology (ARO). For each gene, CARD provided standardized information on the AMR gene family, associated drug class, molecular resistance mechanism, and curated ARO accession numbers, enabling consistent comparison across isolates and sputum resistomes.

### 2.7. Statistical Analysis

Concordance between MALDI-TOF and tNGS results in sputum samples and bacterial cultures was evaluated using the concordance rate and Cohen’s kappa coefficient (K) [37]. As neither method was considered a “gold standard,” diagnostic sensitivity and specificity were not calculated [38,39].

## 3. Results

### 3.1. Cohort Characteristics

A total of 153 sputum samples from patients with CAP were collected between October 2022 and November 2023 at three hospitals in Karaganda. After applying the inclusion and exclusion criteria, 48 culture-positive cases were retained for analysis (Table 1).

The study cohort was predominantly male, with a median age in the older adult range, reflecting the typical demographic most vulnerable to CAP. A substantial proportion of patients had hematological malignancies, often alongside pneumonia, underscoring the high-risk profile of this group. The majority of patients were admitted to the hematology and surgical departments, with a smaller fraction admitted to pulmonology, medicine, cardiology, and intensive care. Clinical outcomes were generally favorable, with most patients discharged; however, three deaths occurred, reflecting the severity of CAP in immunocompromised and comorbid populations.

### 3.2. Identification of K. pneumoniae Using MALDI-TOF MS and tNGS

We compared MALDI-TOF MS and tNGS for *K. pneumoniae* detection in 48 culture-positive CAP cases. MALDI-TOF MS and tNGS were applied to pure bacterial cultures, while tNGS was additionally performed on unprocessed sputum samples (Table 2).

tNGS of culture samples identified *K. pneumoniae* in 22/48 cases (45.8%), MALDI-TOF MS detected 17/48 (35.4%), and tNGS of sputum detected 14/48 (29.2%). Eight cases (16.7%) were concordant across all methods. The highest yield was achieved with tNGS in culture samples, while the lower detection rate in sputum likely reflected matrix complexity, lower pathogen load, and a higher background microbiota. Overall, *K. pneumoniae* was detected in 26/48 (54.2%) samples by at least one method.

#### 3.2.1. Microbial Co-Detection and Diversity

**Figure 1:**
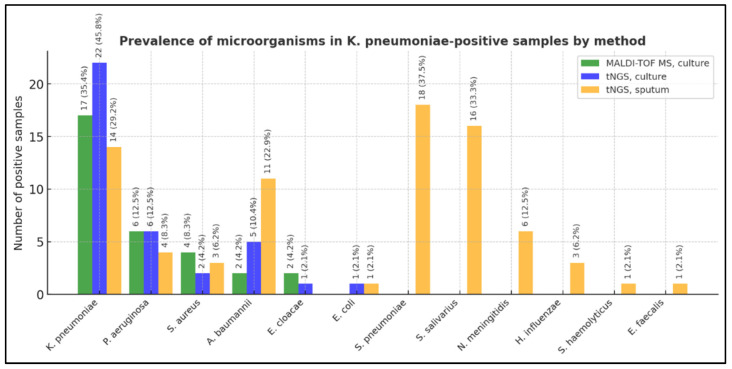
Prevalence of additional microorganisms in samples positive for *K. pneumoniae*. Data are presented by method (MALDI-TOF MS, tNGS of culture, tNGS of sputum).

In *K. pneumoniae*-positive samples, co-pathogens were identified with varying frequency depending on the method used (Table 3, Figure 1). MALDI-TOF MS of culture samples detected five distinct species (mean 1.15 microorganisms/sample), primarily representing dominant isolates due to culture selection. tNGS of culture samples revealed six species (mean 1.42 microorganisms/sample), including *E. coli* co-detected with *K. pneumoniae*.

Direct tNGS analysis of sputum identified 11 species (mean 3.04 microorganisms/sample) and uniquely detected *S. pneumoniae*, *S. salivarius*, *N. meningitidis*, *H. influenzae*, *S. haemolyticus*, and *E. faecalis*, which were absent in the culture results. These findings suggest that molecular profiling of unprocessed sputum can capture a broader range of respiratory taxa, including organisms or their non-viable remnants, which may be lost during cultivation. Such differences highlight inherent methodological contrasts between culture-dependent and sequencing-based diagnostics.

#### 3.2.2. Concordance Analysis

**Figure 2:**
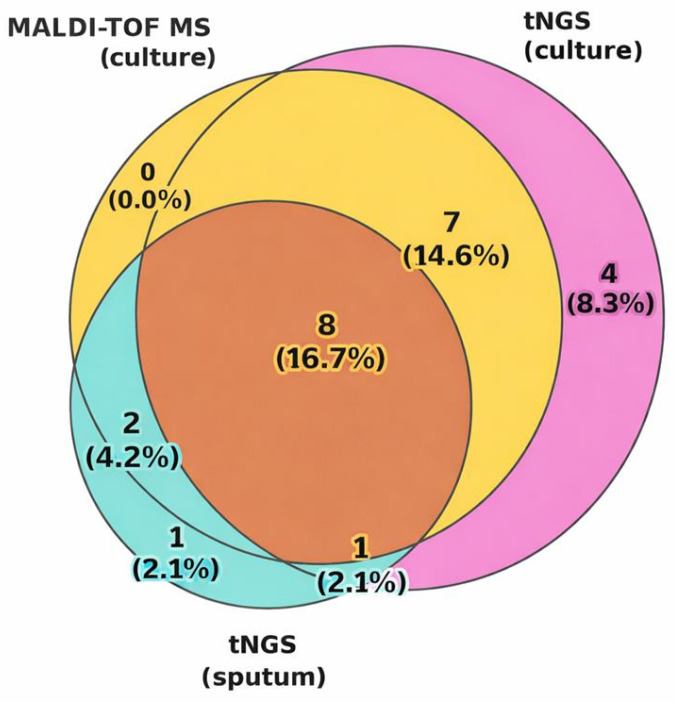
Overlap of *K. pneumoniae* detection between MALDI-TOF MS (culture), tNGS (culture), and tNGS (sputum). Yellow represents MALDI-TOF MS–based identification from cultured isolates, pink represents tNGS performed on cultured isolates, and turquoise represents direct tNGS of sputum samples. Overlapping areas indicate concordant detection between methods. Values indicate the absolute number of positive samples and the percentage relative to the total cohort (*n* = 48).

Agreement between methods is summarized in Table 4 and illustrated in the Venn diagram in Figure 2. The highest overlap was observed between MALDI-TOF MS and tNGS conducted on culture samples (*n* = 15), with eight samples (16.7%) tested positive by all three approaches. Concordance between MALDI-TOF MS and tNGS (culture) was 85.4% (K = 0.712; *p* = 1.05 × 10^−6^), indicating substantial agreement. Comparison of tNGS (culture) vs. tNGS (sputum) and MALDI-TOF MS (culture) vs. tNGS (sputum) showed concordance rates of 64.6% (K = 0.279; *p* = 0.029) and 72.9% (K = 0.383; *p* = 0.0094), respectively, reflecting the influence of sample type on detection rates.

The Venn diagram (Figure 2) highlights that a notable proportion of detections were method-specific, underscoring differences in analytical sensitivity, spectrum of detectable microorganisms, and potential loss of certain pathogens during cultivation.

### 3.3. Comparative Analysis of K. pneumoniae AMR in Sputum and Cultural Samples

#### 3.3.1. Phenotypic Antimicrobial Resistance Profiles

To eliminate the influence of other pathogens, twelve *K. pneumoniae* isolates, confirmed by both MALDI-TOF MS and tNGS, were included in the analysis (Table S1).

**Figure 3:**
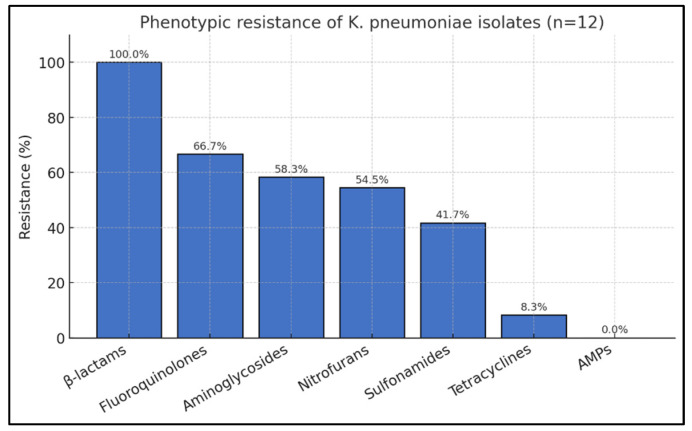
Distribution of phenotypic antimicrobial resistance among 12 *K. pneumoniae* isolates by antibiotic class.

The results demonstrated that all isolates (100%) exhibited partial resistance to the tested β-lactams, which showed the highest resistance rate among all antibiotic classes. This was followed by fluoroquinolones (66.7%), aminoglycosides (58.3%), nitrofurans (54.5%), sulfonamides (41.7%), and tetracyclines (8.3%), while no resistance to antimicrobial peptides was observed. These findings are summarized in Figure 3, which illustrates the distribution of resistance rates across antibiotic classes.

Classification by resistance status revealed four isolates (33.3%) with AMR, four (33.3%) with MDR, and four (33.3%) with XDR phenotypes. Due to incomplete data for two isolates (IDs 17 and 59), the distribution may be slightly underestimated. Overall, two-thirds of the isolates (66.6%) demonstrated MDR or XDR phenotypes, indicating progressive accumulation of resistance mechanisms and severely limited therapeutic options for *K. pneumoniae*-associated pneumonia.

#### 3.3.2. Genotypic–Phenotypic Correlation of Antimicrobial Resistance in Pure Cultures

**Figure 4:**
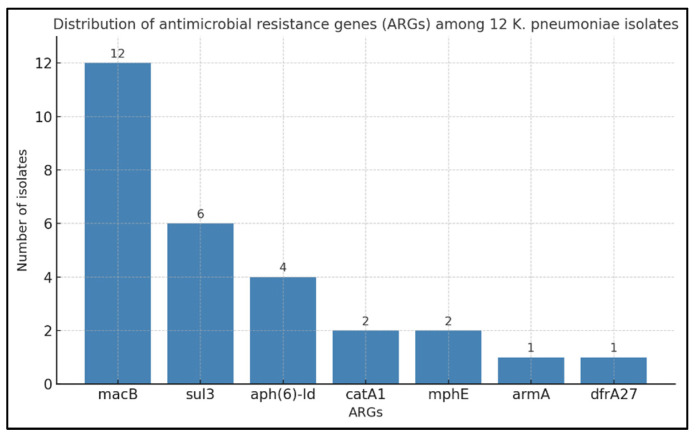
Distribution of ARGs among 12 *K. pneumoniae* isolates confirmed by both MALDI-TOF MS and tNGS.

tNGS of pure cultures identified seven ARGs: *macB*, *sul3*, *mphE*, *aph(6)-Id*, *armA*, *catA1*, and *dfrA27* (Table S1, Figure 4). Among them, *macB* was universal, detected in all 12 isolates (100%). According to CARD, *macB* (ARO:3000535) belongs to the ATP-binding cassette (ABC) antibiotic efflux pump gene family, is associated with macrolide antibiotics, and mediates resistance through antibiotic efflux. This finding is consistent with the intrinsic macrolide resistance of *K. pneumoniae*, mediated by active efflux through the MacAB–TolC system. It aligns with EUCAST recommendations that phenotypic testing for macrolides in *Enterobacterales* is unnecessary [40].

The second most frequent ARG, *sul3*, was detected in six isolates (50%). According to CARD (ARO:3000413), *sul3* encodes a sulfonamide-resistant dihydropteroate synthase that is functionally similar to *sul1* and *sul2* and is typically plasmid-borne; it was originally described in *E. coli* isolates resistant to sulfonamides. Phenotypic sulfonamide resistance was observed in 41.7% of isolates, with concordance between genotype and phenotype in three cases (ID 12, 46, 51). In the remaining isolates (ID 22, 24, 59), the gene was present without phenotypic resistance, possibly reflecting low expression, compensatory metabolic pathways, or other uncharacterized mechanisms.

*Aph(6)-Id*, encoding aminoglycoside phosphotransferase, was found in four isolates (ID 22, 46, 51, 59). According to CARD (ARO:3002660), *aph(6)-Id* confers resistance to aminoglycoside antibiotics and is typically encoded on plasmids, integrative conjugative elements, and chromosomal genomic islands in *K. pneumoniae*, reflecting its mobility and potential for horizontal gene transfer. Three of these cases exhibited phenotypic aminoglycoside resistance, while one (ID 22) did not. At the same time, four other resistant isolates lacked *aph(6)-Id* (ID 17, 24, 39, 42), suggesting alternative resistance mechanisms, such as other aminoglycoside-modifying enzymes, ribosomal mutations, or efflux pumps. Of note, isolate ID 24 carried *armA* (ARO:3000858), encoding a 16S rRNA methyltransferase responsible for high-level aminoglycoside resistance.

Genes *mphE* (ARO:3003741), *catA1* (ARO:3002683), and *dfrA27* (ARO:3004550) were found in one or two isolates and also showed partial correlation with phenotypic resistance. Phenotypic testing for macrolides and chloramphenicol was not performed because *K. pneumoniae* is intrinsically resistant to these classes per EUCAST guidelines. In contrast, trimethoprim resistance testing was not conducted for the single *dfrA27*-positive isolate (ID 46).

To summarize the distribution of detected ARGs, all seven genes were grouped by their corresponding antibiotic classes according to CARD annotations. Macrolide-associated determinants included *macB* (12 isolates) and *mphE* (two isolates); aminoglycoside-related genes comprised *aph(6)-Id* (four isolates) and *armA* (one isolate). Additional ARGs were *sul3* (six isolates; sulfonamides), *catA1* (two isolates; phenicols), and *dfrA27* (one isolate; diaminopyrimidines). Comparison with phenotypic AST showed only partial agreement. For example, sulfonamide and aminoglycoside resistance correlated with the presence of *sul3, aph(6)-Id*, or *armA* in several isolates. In contrast, the phenotypically observed universal β-lactam resistance was not supported by β-lactamase genes in tNGS data, suggesting the presence of alternative mechanisms (Table S1).

Taken together, the genotype–phenotype discrepancies observed here, along with the inherent limitations of targeted tNGS, highlight the need for cautious clinical interpretation. Beyond incomplete ARG coverage, tNGS cannot detect non-genetic resistance mechanisms (e.g., efflux regulation and changes in membrane permeability). At this stage, such panels remain premature for standalone clinical use and should complement, not replace, phenotypic AST to ensure reliable antimicrobial decision-making in *K. pneumoniae* CAP.

#### 3.3.3. Comparative Analysis of ARG Profiles in Sputum and Pure *K. pneumoniae* Cultures

**Figure 5:**
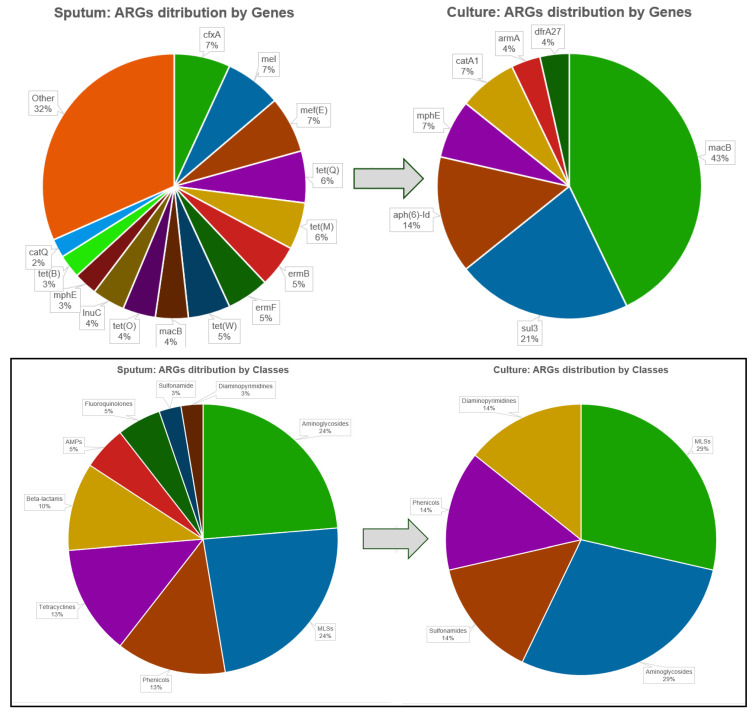
Comparative distribution of ARGs detected by tNGS in pure *K. pneumoniae* cultures (*n* = 12) and corresponding sputum samples from CAP patients. Upper panel: distribution of ARGs by individual genes. Lower panel: distribution of ARGs by antibiotic classes. The arrow highlights structural shifts in the resistome composition between sputum and pure culture analysis.

A comparative tNGS analysis of ARGs in pure *K. pneumoniae* isolates and matched primary sputum samples from CAP patients revealed pronounced differences in resistome composition (Table S2, Figure 5). Across 12 paired samples, only 7 distinct ARGs were detected in cultures, compared with 38 in sputum, yielding mean values of 2.3 and 14.3 ARGs per sample, respectively. This >6-fold increase highlights the contribution of the broader respiratory microbiome to the “clinical resistome,” with many resistance determinants lost during cultivation.

The distribution of ARG patterns differed substantially between cultures and between sputum samples. In cultures, *macB* was universal (12/12, 100%), followed by *sul3* (50%), *aph(6)-Id* (33%), *catA1* (17%), *mphE* (17%), *armA* (8%), and *dfrA27* (8%). By contrast, sputum samples were dominated by *cfxA*, *mel*, and *mef(E)*, all present in 100% of cases, followed by *tet(Q)* (91.6%), *tet(M)* (83.3%), *ermB*, *ermF*, and *tet(W)* (75%), while *macB* was found in only seven sputum samples (58.3%).

By antibiotic classes, pure cultures were limited mainly to MLSs (*macB*, *mphE*), aminoglycosides (*aph(6)-Id*, *armA*), and a few single determinants for sulfonamides (*sul3*), phenicols (*catA1*), and diaminopyrimidines (*dfrA27*). In contrast, sputum-derived resistomes included 9 ARGs for MLSs, 9 for aminoglycosides, 5 for phenicols, 5 for tetracyclines, 4 for β-lactams, 2 for antimicrobial peptides, 2 for fluoroquinolones, and one each for sulfonamides and diaminopyrimidines.

Interestingly, in several cases (ID 5, 18, 24, 46, 51, 59), ARGs were present in cultures but absent from the corresponding sputum samples, likely reflecting the dependence of these genes on *K. pneumoniae* itself. For example, isolate ID 51 harbored *aph(6)-Id*, *macB*, and *sul3* in culture, none of which were found in the matched sputum, where *K. pneumoniae* was undetected. Such cases strongly suggest that these ARGs were carried by *K. pneumoniae* rather than by the broader microbial community.

### 3.4. Interesting Findings That Warrant Further Investigation

#### 3.4.1. Divergent Microbial and Resistome Profiles in Fatal CAP Cases

Two fatal cases (ID 3 and ID 32) exhibited notable differences between culture-based and metagenomic findings, illustrating how methodological variation can influence the characterization of microbial communities and associated resistance determinants (Table S3).

*Case 1: ID 3 (45-year-old male, levofloxacin therapy, death)*

Culture identified *P. aeruginosa* (19,969 reads) with a relatively narrow resistome (five ARGs, including aminoglycoside phosphotransferases and *catB7*). In contrast, sputum tNGS revealed a polymicrobial community—*K. pneumoniae* (51,718 reads), *A. baumannii* (12,686 reads), *E. faecalis*, and *S. pneumoniae*—and a markedly broader resistome. Additional determinants of clinical concern included *armA* (856 reads), *vanY* (830 reads), *eptA* (819 reads), and macrolide efflux genes (*mef(E)*, *mel*). These differences likely reflect the inherent analytical distinctions between cultivation, which selects for readily growing organisms, and direct sequencing, which captures a wider range of detectable DNA.

*Case 2: ID 32 (60-year-old male, multiple prior antibiotics, death)*

Culture results were dominated by *A. baumannii* (645,296 reads) and yielded only one ARG (*uppP*, 59,962 reads). Direct sputum tNGS, however, identified co-dominant *A. baumannii* (55,081 reads) and *K. pneumoniae* (52,725 reads), with a total of 16 distinct ARGs. These included high-level aminoglycoside resistance genes (*armA*, *aph(3’)-VIa*, *ant(6)-Ia*), macrolide resistance genes (*mphE*, *mef(E)*, *mel*), and additional determinants (*dfrA5*, *catA1*, *cfxA*, *eptA*, *uppP*). The absence of these taxa and genes in culture may reflect differential growth dynamics, selective enrichment, or reduced viability of certain organisms under standard cultivation conditions.

#### 3.4.2. Divergent Culture- and Sputum-Based Profiles in CAP Patients

*Case 3: ID 5 (81-year-old male, multiple myeloma, discharged)*

Sputum tNGS identified *S. pneumoniae* (85,726 reads) and *N. meningitidis* (15,406 reads) as dominant taxa, while *K. pneumoniae* was detected only at a sub-threshold level (29 reads) (Table S3). The sputum resistome contained 12 ARGs, including macrolide efflux determinants (*mef(E)*, *mel*), both macrolide efflux determinants co-expressed in *S. pneumoniae* [41], ribosomal methylase (*ermB*), multiple tetracycline resistance genes (*tet(M)*, *tet(O)*, *tet(Q)*, *tet(W)*), and *cfxA*. After cultivation, however, only *K. pneumoniae* (163,856 reads) was recovered, carrying a single ARG, *macB* (27,809 reads). This shift in the detected microbial community—from domination by *S. pneumoniae* in sputum to sole recovery of *K. pneumoniae* in culture—highlights how cultivation and sequencing may capture different aspects of the sample, reflecting growth-dependent versus DNA-based detection.

*Case 4: Consistent Loss of S. pneumoniae-Associated ARGs*

This phenomenon was observed in multiple samples (IDs 18, 24, 46, 49, 51, 59; Table S3).

- ID 18: Loss of *mef(E)* and *pbp1A*, a penicillin-binding protein in *S. pneumoniae* that confers resistance to penicillin-class β-lactams and cephalosporins [42].
- ID 24: Loss of *mel*.
- IDs 46, 49, 51, 59: Loss of both *mel* and *mef(E)*.

In all these cases, *S. pneumoniae* was detected in sputum by tNGS but not in pure cultures. These observations likely reflect differences in growth behavior or viability, as well as the ability of sequencing to detect DNA from organisms that may not proliferate under standard cultivation conditions.

*Case 5: Detection of mecA in Sputum but Not in Culture*

The *mecA* gene was detected in sputum samples from IDs 18, 46, and 51 (Table S3). *mecA* is associated with methicillin resistance in staphylococci, including *Staphylococcus aureus*, conferring resistance to methicillin and other β-lactams (e.g., penicillin, oxacillin, amoxicillin) by expressing the penicillin-binding protein 2a (PBP2a) [43,44]. The detection of *mecA* only in sputum suggests the presence of low-abundance or non-viable MRSA-related genetic material, reinforcing that tNGS of culture samples may reveal complementary aspects of the microbial and resistome profiles.

## 4. Discussion

In this pilot study, we investigated the prevalence of *K. pneumoniae* in patients with CAP, compared diagnostic outcomes between primary sputum and corresponding bacterial cultures, and evaluated the performance of conventional methods alongside tNGS. The results revealed substantial differences between molecular and culture-based approaches in pathogen detection, microbial diversity, and ARG profiles.

The following discussion is organized into several subsections to contextualize our results within the broader clinical and epidemiological landscape.

### 4.1. Prevalence and Clinical Importance of K. pneumoniae

*K. pneumoniae*, a major Gram-negative pathogen first described by Friedländer in 1882 [45], is a frequent cause of both CAP and HAP. While in most Western epidemiological studies its CAP prevalence rarely exceeds 15% [46,47], in our cohort *K. pneumoniae* was detected in 14% of all patients (22/153), fully consistent with international data. At the same time, its contribution among culture-positive cases was disproportionately high, reaching 54% (22/48).

In contrast, a previous national study of COVID-associated pneumonia in Kazakhstan reported a higher prevalence of *K. pneumoniae* (23%), suggesting possible differences in study populations, clinical settings, or pathogen distribution between CAP and COVID-related cohorts. That study was also limited by a relatively small sample size (209 patients) and, unlike our work which was restricted to Karaganda, encompassed a broader geographical area including Almaty, Karaganda, and Atyrau, Kazakhstan [8]. This divergence underscores the context-dependent role of *K. pneumoniae* in respiratory infections and highlights the need for continuous epidemiological surveillance across diverse patient groups and regions.

Methodological comparisons revealed detection rates of 45% by tNGS of cultures, 35% by MALDI-TOF MS, and 29% by direct sputum tNGS. Only 16% of cases were concordant across all three methods. The highest agreement was between MALDI-TOF MS and tNGS on cultures (κ = 0.712, *p* = 1.05 × 10^−6^), whereas concordance between sputum and culture tNGS was significantly lower (κ = 0.279, *p* = 0.029). This discrepancy highlights the challenges of comparing single-isolate analyses with complex polymicrobial communities, which may also be affected by PCR inhibitors present in respiratory samples [48,49].

### 4.2. Fatal Outcomes and Diagnostic Discordance

Among 153 CAP patients, three fatal outcomes were documented (6.2% of culture-positive cases). In two of these cases, sputum tNGS identified *K. pneumoniae* and a broader set of ARGs. At the same time, culture-based diagnostics recovered only *P. aeruginosa* or *A. baumannii* with more limited resistome profiles. These patterns reflect methodological differences: cultivation depends on organism viability and growth dynamics, whereas tNGS captures a wider range of microbial DNA, including minority or fastidious taxa. Overall, these findings illustrate that culture-based methods may provide a more limited view of microbial diversity and resistance determinants. In contrast, tNGS can detect additional taxa and ARGs that are not recovered in culture. At the same time, tNGS identifies DNA from both viable and non-viable microorganisms; thus, some sputum-derived signals may not represent active contributors to infection. These observations underscore the importance of integrating molecular and culture-based data and highlight the need for future studies that incorporate viability assays or complementary molecular approaches to better contextualize discordant findings.

### 4.3. Microbial Diversity and Cultivation Effects

Direct tNGS of primary sputum revealed significantly greater microbial diversity than culture-based approaches. While MALDI-TOF MS and tNGS of culture samples detected an average of 1.15 and 1.42 species per sample, respectively, sputum tNGS identified 11 taxa in total and an average of 3.04 species per sample. Six clinically relevant microorganisms—*S. pneumoniae*, *S. salivarius*, *N. meningitidis*, *H. influenzae*, *S. haemolyticus*, and *E. faecalis*—were only detected by sputum tNGS and were completely absent from culture results, suggesting that cultivation preferentially supports dominant or fast-growing species while suppressing others [50,51,52]. At the same time, it should be acknowledged that tNGS also has inherent limitations, as it detects DNA from both viable and non-viable organisms, which may lead to overestimation of microbial diversity and resistome complexity.

Polymicrobial infections are common in pneumonia [53], occurring in up to 76.4% of pediatric [54] and 11.2% of adult [55] influenza-associated cases. In our dataset, sputum tNGS tended to reveal greater microbial complexity, whereas culture methods generally produced simpler community profiles. We also noted both the “loss” and “appearance” of *K. pneumoniae* between sputum and culture, which may result from interspecies competition [56,57], viable but non-culturable states [58,59], suboptimal media or incubation parameters [60], or prior antibiotic exposure [61]. Variability in sputum quality, viscosity, and potential oropharyngeal contamination [62,63] can further distort results.

### 4.4. Antimicrobial Resistance Patterns

In this pilot study, both phenotypic (DDM) and molecular (tNGS) approaches were applied to characterize the AMR profiles of *K. pneumoniae*, enabling direct comparison of resistance expression with underlying genetic determinants [45,64]. Such integration is essential for understanding the prevalence, mechanisms, and clinical implications of AMR in this high-priority pathogen.

Our phenotypic data revealed an alarming situation: all isolates were resistant to tested β-lactams except carbapenems, most likely due to the near-universal presence of extended-spectrum β-lactamases (ESBLs) in hospital-associated *K. pneumoniae*. This finding effectively eliminates cephalosporins and other β-lactams as viable empirical options, shifting therapeutic reliance to “last-resort” drugs [65]. Carbapenem-resistant *K. pneumoniae* (CRKP) has been designated by the WHO as a critical-priority pathogen [66,67], and its detection in 41.6% of our isolates, with the majority showing cross-resistance, strongly suggests the active circulation of carbapenemase-producing strains [68]. These results mirror global trends, such as CHINET surveillance data reporting a rise in carbapenem resistance in China from ~3% in 2005 to over 20% by 2017 [69].

Comparable challenges have been documented in Kazakhstan. Recent local studies reported high rates of ESBL-producing *K. pneumoniae* and an increasing prevalence of multidrug-resistant and carbapenem-resistant strains in both hospital and community settings, particularly during the COVID-19 pandemic. For example, in a multicenter study of patients with “COVID-like pneumonia,” *K. pneumoniae* was the most frequently detected bacterial pathogen (23%), and 68% of isolates demonstrated phenotypic ESBL production [8]. Another retrospective analysis of 169 *K. pneumoniae* isolates obtained from multiple biological materials confirmed high resistance levels across several antibiotic classes, while identifying imipenem (84.5%), minocycline (82.4%), and amikacin (79.6%) as the most effective agents, with resistance rates generally below 20% [70]. Genotypic analysis by tNGS covered more than 110 ARGs associated with β-lactam and carbapenem resistance, including *blaCTX-M*, *blaTEM*, *blaSHV*, *blaKPC*, *blaNDM*, *blaVIM*, *blaOXA-48-like*, and *blaIMP*, yet none were detected in our isolates. This absence may be attributable to alternative resistance mechanisms or to the inherent limitations of the targeted tNGS panel [71,72]. Co-resistance to other antibiotic classes was common, with high rates for fluoroquinolones (66.7%) and aminoglycosides (58.3%), and moderate rates for nitrofurans (54.5%) and sulfonamides (41.7%). Despite the presence of 16 fluoroquinolone resistance genes (*qnr*, *oqxAB*) in the panel, none were detected, suggesting chromosomal mutations in *gyrA* and *parC* IV [73]. Key nitrofuran resistance genes (*nfsA*, *nfsB*, *ribE*) were also absent. Aminoglycoside resistance was partly explained by *APH(6)-Id* [74] and *armA* [74,75,76], though concordance with phenotype was incomplete. Sulfonamide resistance was linked to *sul3* [77] in 50% of isolates, with phenotypic agreement in only half, possibly due to gene regulation, alternative metabolic pathways, or other mechanisms [78]. Phenotypic tetracycline resistance was observed in one isolate, but no corresponding genes (despite 41 panel variants) were detected; no peptide antibiotic resistance was found.

Genotypic analysis identified only seven ARGs in pure cultures: *macB*, *sul3*, *mphE*, *aph(6)-Id*, *armA*, *catA1*, and *dfrA27*. *macB*, detected in all isolates, encodes an ABC transporter associated with intrinsic macrolide resistance [79], consistent with Gram-negative impermeability and EUCAST guidance. *mphE* (*n* = 2) encodes macrolide phosphotransferase [80], *catA1* (*n* = 2) encodes chloramphenicol acetyltransferase [81] (common in *Enterobacterales*, prevalence >50% in some regions [68]), with resistance potentially reversible after drug withdrawal [82]. *dfrA27* (*n* = 1) encodes plasmid-borne dihydrofolate reductase conferring trimethoprim resistance [83].

In summary, the genotype–phenotype discrepancies observed in our isolates, together with the inherent limitations of targeted tNGS, underscore the need for cautious clinical interpretation. Because tNGS captures only a subset of resistance determinants and cannot detect non-genetic mechanisms such as efflux regulation or membrane permeability changes, it is not yet suitable as a standalone tool. Instead, targeted sequencing should complement, rather than replace, classical culture-based AST, which remains essential for phenotypic confirmation and MIC-based therapeutic decision-making in *K. pneumoniae* CAP.

Overall, two-thirds of our isolates were classified as MDR or XDR, with equal distribution across the AMR, MDR, and XDR categories (33.3% each), reflecting a progressive trend toward greater resistance complexity. The convergence of universal β-lactam resistance, high CRKP prevalence, and notable co-resistance patterns in *K. pneumoniae* poses a substantial challenge for clinical management. These observations highlight the importance of stringent infection control measures, optimized antimicrobial stewardship, and continued efforts to develop novel therapeutic strategies.

### 4.5. Resistome Profiles in Cultures Versus Primary Sputum

This pilot study presents a direct comparison of ARG profiles from *K. pneumoniae* pure cultures and corresponding primary sputum samples of CAP patients using tNGS. We observed a >6-fold increase in the number and diversity of ARGs in sputum (38 unique genes) versus cultures (seven genes), highlighting the fundamental difference between analytical targets: sputum represents the complex respiratory microbiome [84,85,86], whereas pure cultures capture only the genome of a single in vitro proliferating organism [87].

Many sputum-specific ARGs, including tetracycline and β-lactam determinants, are absent in cultures and likely originate from commensals or uncultivable/slow-growing pathogens, contributing to pneumonia’s polymicrobial etiology [88]. Dominant ARGs also differed: *macB* was consistently present in cultures, whereas sputum resistomes were dominated by *cfxA, mel*, and *mef(E)*—often linked to anaerobes such as *S. pneumoniae* [41,89]. These findings suggest that the resistome is shaped not only by dominant cultivable pathogens but also by the broader microbial community, with potential implications for horizontal transfer of ARGs and their impact on infection dynamics and treatment outcome [90].

### 4.6. Microbial Shifts Between Sputum and Culture

An illustrative example is provided by sample ID 5. In the primary sputum specimen, *N. meningitidis* and *S. pneumoniae* were the dominant taxa, whereas *K. pneumoniae* was present only in trace amounts (29 reads), falling below the detection threshold for standard analysis. After cultivation, however, *K. pneumoniae* was the sole recovered species, and the initially dominant taxa were not detected. This was accompanied by the disappearance of all ARGs initially observed in the sputum and the appearance of *macB*, presumably associated with *K. pneumoniae*.

Comparable patterns were observed in other cases (IDs 18, 24, 46, 49, 51, 59), where cultivation supported the outgrowth of *K. pneumoniae* even when it was initially present at low abundance. The loss of *S. pneumoniae* in several cases coincided with the absence of its characteristic resistance determinants, including *mel*, *mef(E)*, and *pbp1A.* [41,42]. These observations suggest that culture conditions may favor particular taxa, thereby influencing which resistance genes are ultimately represented. At the same time, tNGS of sputum provides a broader view of the microbial and resistome diversity present at the infection site.

### 4.7. Detection of Clinically Significant but Hidden Resistance Markers

A particularly noteworthy finding of this pilot study is the detection of the *mecA* gene in three primary sputum samples (ID 8, 46, 51). This gene is a hallmark marker of methicillin-resistant *S. aureus* (MRSA), a pathogen posing a serious public health threat [91]. The presence of MRSA in patients’ sputum is critical for guiding antimicrobial therapy, as timely recognition could significantly influence treatment outcomes. However, *mecA* was absent from the corresponding culture-derived samples, in which *K. pneumoniae* predominated. This stark contrast highlights a major clinical risk associated with reliance on culture-based diagnostics: important pathogens and their resistance determinants can be entirely overlooked when workflows are designed to recover only the dominant cultivable species.

While tNGS can reveal clinically relevant markers missed by culture, including *mecA* and other ARGs detectable directly in sputum, its interpretation must account for key limitations. Targeted panels assess only predefined loci, cannot determine organism viability, and may detect residual DNA unrelated to active infection. These constraints may inflate perceived diversity or resistome complexity. Thus, tNGS should be viewed as a complementary tool, whereas culture-based identification and phenotypic AST remain essential for verifying active pathogens and informing targeted therapy in CAP.

Incorporating molecular methods such as tNGS into diagnostic workflows has the potential to markedly improve respiratory infection diagnostics, including CAP. Reported sensitivities reach 92.6%, surpassing those of conventional methods [92]. Beyond pathogen detection, tNGS enables simultaneous identification of virulence factors and resistance genes [23], which, in clinical practice, has been associated with reduced mortality and shorter durations of mechanical ventilation [92]. Yet, broad implementation remains limited by high costs, specialized infrastructure, and the need for trained personnel [92]. Moreover, genotypic resistance does not always align with phenotypic susceptibility, with concordance rates averaging around 65% [24].

Therefore, the future of CAP diagnostics lies in integrating culture-based and molecular approaches. Achieving this will require user-friendly analytical platforms capable of translating genomic data into actionable reports [93], thereby optimizing therapy, reinforcing infection control, and supporting novel strategies against rising antimicrobial resistance.

### 4.8. Complementary Roles of Classical Microbiological Methods and tNGS in CAP

The discrepancies observed between culture-based and molecular findings in this pilot study underscore the fundamentally different analytical targets of conventional microbiology versus sequencing-based approaches. Classical diagnostics, comprising cultivation, MALDI-TOF MS identification, and DDM, remain the cornerstone of clinical decision-making, as they provide viable isolates and phenotypic susceptibility profiles indispensable for antimicrobial therapy. However, these methods inherently depend on organism growth, selectively recovering taxa that thrive under laboratory conditions and potentially overlooking fastidious, slow-growing, or suppressed members of polymicrobial communities [94].

In contrast, tNGS analyzes DNA directly from sputum or cultured isolates and can therefore detect a wider range of microorganisms, including fastidious, non-cultivable, and low-abundance taxa. The broader diagnostic value of targeted sequencing has also been recognized beyond pneumonia, for example, the World Health Organization (WHO) recommends its use to improve tuberculosis diagnostics and guide earlier, more effective treatment, particularly in resource-limited settings [95]. This methodological difference explains several key findings of our pilot study, including the higher microbial diversity and significantly expanded resistome detected in sputum compared with pure cultures. At the same time, the panel’s targeted scope and its inability to distinguish viable from non-viable cells remain important limitations. Although methods such as propidium monoazide (PMA) treatment or RNA-based sequencing can address this issue [96,97], they were not applied in our study and should be considered in future work.

To contextualize these methodological differences and guide their practical integration into clinical workflows, we provide a comparative summary of the diagnostic characteristics of the techniques used in this pilot study (Table 5). The table outlines their turnaround times, detection capabilities, strengths, and limitations, emphasizing how each method contributes unique and complementary information to the diagnostic process.

Thus, rather than replacing traditional methods, tNGS should be regarded as a valuable adjunct that enhances diagnostic precision, supports informed antimicrobial stewardship, and contributes to a more comprehensive understanding of respiratory infection biology.

## 5. Conclusions

This pilot study highlights the significant role of *K. pneumoniae* in CAP and demonstrates that culture-based diagnostics and tNGS capture distinct yet complementary aspects of its detection and resistance profiling. Direct sputum tNGS revealed greater microbial diversity and a broader resistome than pure *K. pneumoniae* cultures. In contrast, classical methods remain essential for species confirmation, phenotypic susceptibility testing, and clinical decision-making. Importantly, tNGS complements but does not replace culture-based workflows; its targeted design and inability to distinguish viable from non-viable organisms necessitate cautious interpretation. An integrated diagnostic strategy—combining culture, MALDI-TOF MS, DDM, and tNGS, with future incorporation of viability-discriminating approaches—offers the most robust framework for improving pathogen detection, guiding antimicrobial therapy, and strengthening AMR surveillance in *K. pneumoniae*–associated CAP.

## 6. Limitations of This Study

This pilot study is limited by the inclusion criterion requiring culture growth of a presumptive CAP pathogen, which may bias results toward cultivable bacteria and underrepresent fastidious or non-cultivable species detectable only by molecular methods. In addition, the use of the Ion AmpliSeq™ Pan-Bacterial Research Panel—a targeted rather than shotgun metagenomic approach—restricts detection to taxa and ARGs included in the primer design, potentially underestimating both the “clinical resistome” and the “isolate resistome.” This limitation underscores the need for validation using broader molecular methods, including PCR or shotgun metagenomics.

Another limitation is that standard tNGS cannot differentiate between DNA from viable and non-viable microorganisms. Prior antibiotic exposure or immune clearance may therefore account for some of the discrepancies between culture and molecular results, as non-viable taxa can still contribute ARGs to the “clinical resistome.”

Furthermore, statistical testing of AMR data was not performed due to the relatively small cohort size, the rarity of certain resistance mechanisms, and the multicomponent nature of some cases. Instead, AMR findings are presented descriptively through figures and tables to provide transparency while avoiding overinterpretation.

Future studies should include larger, multicenter cohorts, encompass diverse pneumonia etiologies and viral co-pathogens, and integrate culture, molecular, and viability assays to improve diagnostic accuracy, antimicrobial stewardship, and infection-control strategies.

## Figures and Tables

**Table 1:** Distribution of patient characteristics (*n* = 48).

Characteristics	*n* (%)
**Sex**	
Male	32 (66.7)
Female	16 (33.3)
**Age**	
Median (IQR)	67.0 (55–75)
Mean (range)	63.4 (24–90)
**Institution**	
Clinic at the Medical University	31 (64.6)
Hematology Center	14 (29.2)
Cardiology Center	3 (6.2)
**Department**	
Hematology	17 (35.4)
Surgery	15 (31.3)
Pulmonology	8 (16.7)
Medicine	4 (8.3)
Cardiology	3 (6.3)
ICU	1 (2.1)
**Primary Diagnosis Categories**	
CAP only	15 (31.3)
Hematological malignancy + pneumonia	21 (43.7)
Other conditions + pneumonia	12 (25.0)
**Specific Hematological Malignancies**	
Acute myeloid leukemia	6 (12.5)
Multiple myeloma	6 (12.5)
Myelodysplastic syndromes	3 (6.3)
Chronic lymphocytic leukemia	2 (4.2)
Other leukemias	4 (8.3)
**Clinical Outcomes**	
Discharged	45 (93.8)
Death	3 (6.2)

**Table 2:** Detection of *K. pneumoniae* by MALDI-TOF MS and tNGS in pure bacterial culture and unprocessed sputum samples. Values are given as absolute numbers and percentages of the total cohort (*n* = 48).

Method	Positive Samples (*n*)	% of Total (*n* = 48)
MALDI-TOF MS, culture	17	35.4
tNGS, culture	22	45.8
tNGS, sputum	14	29.2
All methods	8	16.7
Any method	26	54.2

Note: “Culture”—pure bacterial culture samples; “Sputum”—unprocessed sputum samples. “All methods”—cases where *K. pneumoniae* was detected by all three approaches. “Any method”—cases where *K. pneumoniae* was detected by at least one method.

**Table 3:** Co-detection of additional microorganisms in *K. pneumoniae*-positive samples. Values indicate the number of samples in which each species was detected. Species marked with ^1^ were found exclusively in sputum samples by tNGS.

Microorganisms	MALDI-TOF MS, Culture	tngs, Culture	tngs, Sputum
**Gram-negative**			
*Pseudomonas aeruginosa*	6	6	4
*Acinetobacter baumannii*	2	5	11
*Enterobacter cloacae*	2	1	-
*Escherichia coli*	-	1	1
*Haemophilus influenzae* ^1^	-	-	3
**Gram-positive**			
*Staphylococcus aureus*	4	2	3
*Staphylococcus haemolyticus* ^1^	-	-	1
*Enterococcus faecalis* ^1^	-	-	1
***Streptococcus* spp.**			
*Streptococcus pneumoniae* ^1^	-	-	18
*Streptococcus salivarius* ^1^	-	-	16
**Other**			
*Neisseria meningitidis* ^1^	-	-	6
**Totals**			
**Number of distinct species**	**5**	**6**	**11**
**Mean microorganisms/sample**	**1.15**	**1.42**	**3.04**

**Table 4:** Concordance rates and Cohen’s kappa coefficients for pairwise comparison of *K. pneumoniae* detection between MALDI-TOF MS (culture), tNGS (culture), and tNGS (sputum).

Comparable Methods	Concordance Rate, % (K)
MALDI-TOF MS and tNGS (culture)	85.4 (K = 0.712; *p* = 1.05 × 10^−6^)
tNGS (culture) and tNGS (sputum)	64.6 (K = 0.279; *p* = 0.029)
MALDI-TOF MS and tNGS (sputum)	72.9 (K = 0.3834; *p* = 0.0094)
All three methods	31.2

**Table 5:** Complementary roles of classical culture and tNGS in CAP.

Parameter	Cultivation + MALDI-TOF MS + DDM (AST)	tNGS
Turnaround time	72–144 h	24–48 h
Detects	Viable bacteria; dominant isolate; phenotypic resistance	DNA from viable + non-viable organisms; predefined taxa and ARGs
Strengths	Species confirmation; phenotypic AST; MIC-based therapy; recovery of isolates	Early broad detection; polymicrobial communities; fastidious organisms; ARG identification
Limitations	Requires growth; may miss minority/fastidious species; slower turnaround	Cannot assess viability; limited to panel targets; genotype–phenotype gaps; requires sequencing infrastructure
Optimal use	Definitive diagnosis; AST-guided therapy; epidemiology	Early pathogen/ARG detection; culture-negative cases; polymicrobial and pretreated infections
Role in CAP management	Essential for final confirmation and susceptibility	Complementary tool improving early decision-making and stewardship

Note: AST—antimicrobial susceptibility testing; MIC—minimum inhibitory concentration. As illustrated in Table 5, classical microbiological workflows and tNGS address different but complementary diagnostic needs. Classical methods remain indispensable for final species identification, MIC-based antimicrobial susceptibility testing, and epidemiological surveillance. Meanwhile, tNGS offers clear advantages in early pathogen detection, characterization of polymicrobial infections, and rapid identification of antimicrobial resistance genes, particularly in cases where prior antibiotic exposure or low bacterial viability complicates culture-based diagnostics.

## Data Availability

The raw data have been submitted to NCBI. The project accession number is PRJNA1165446.

## Supplementary Materials

The following supporting information can be downloaded at https://www.mdpi.com/article/10.3390/diagnostics16010154/s1. Table S1: AMR profiles and associated resistance genes in *K. pneumoniae* isolates; Table S2: tNGS-based identification of ARGs in pure *K. pneumoniae* cultures and primary sputum samples from CAP patients; Table S3: Culture–sputum discrepancies in microbial composition and ARG profiles in CAP.
